# Supplementary material for: Near Perfect Absorber for Long-Wave Infrared Based on Localized Surface Plasmon Resonance
Source: Nanomaterials (Basel). 2022 Nov 27;12(23):4223. doi: 10.3390/nano12234223 (PMC9736474; doi:10.3390/nano12234223)
Supplement: Supplementary file 1 [file nanomaterials-12-04223-s001.zip › nanomaterials-1995586-supplementary.pdf]

nanomaterials

Supplementary materials

Near Perfect Absorber for Long-Wave Infrared Based on Localized Surface Plasmon Resonance

Leihao Sun [1,2], Dingquan Liu [1,2,3,*], Junli Su [1,2], Xingyu Li [2], Sheng Zhou [1], Kaixuan Wang [1,3] and Qiuyu Zhang [1,2]

[1] Shanghai Institute of Technical Physics, Chinese Academy of Sciences, Shanghai 200083, China
[2] School of Physical Science and Technology, ShanghaiTech University, Shanghai 200031, China
[3] School of Optoelectronics, University of Chinese Academy of Sciences, Beijing 100049, China
* Correspondence: dqliu@mail.sitp.ac.cn (D.L.); Tel.: +86-21-25051303; +86-15216701692

S1. Simulation parameters

Simulation temperature-300K

Simulation time－5000fs

Mesh settings：

Mesh Type-auto non-uniform

Mesh accuracy-4

Mesh refinement-conformal variant 0

Boundary conditions：

X-periodic

Y-periodic

Z-Perfect Matched Layer (PML)

PML settings：

Type-stretched coordinate PML

Profile-steep angle layers-12

Source settings：

Source shape-plane wave

Plane wave type-Bloch/periodic

S2. S-parameters method

When we equate a complex structure as a homogeneous 1D slab, the transfer matrix has the analytic form：

$$\vec{\mathrm{T}} = \begin{pmatrix} \cos(nkd) & -z\sin(nkd)/k \\ k\sin(nkd)/z & \cos(nkd) \end{pmatrix}$$

Where n represents its equivalent refractive index, z represents its equivalent impedance, and d is the thickness of the entire structure. The elements of the S matrix can be found from the elements of the T matrix as follows:

$$S_{21} = \frac{2}{T_{11} + T_{22} + (ikT_{12} + \frac{T_{21}}{ik})}$$

$$S_{11} = \frac{T_{11} - T_{22} + (ikT_{12} - \frac{T_{21}}{ik})}{T_{11} + T_{22} + (ikT_{12} + \frac{T_{21}}{ik})}$$

$$S_{22} = \frac{T_{22} - T_{11} + (ikT_{12} - \frac{T_{21}}{ik})}{T_{11} + T_{22} + (ikT_{12} + \frac{T_{21}}{ik})}$$

$$S_{12} = \frac{2\det(\vec{T})}{T_{11} + T_{22} + (ikT_{12} + \frac{T_{21}}{ik})}$$

For a homogeneous 1D slab, *T*11=*T*22=*Ts* and dets **(T)** =1, and the S matrix is symmetric. Thus:

$$S_{21} = S_{12} = \frac{1}{T_s + \frac{1}{2}(ikT_{12} + \frac{T_{21}}{ik})}$$

$$S_{11} = S_{22} = \frac{\frac{1}{2}(\frac{T_{21}}{ik} - ikT_{12})}{T_s + \frac{1}{2}(ikT_{12} + \frac{T_{21}}{ik})}$$

Using the analytic expression for the T-matrix elements gives the S parameters:

$$S_{21} = S_{12} = \frac{1}{\cos(nkd) - \frac{i}{2}(z + \frac{1}{z})\sin(nkd)}$$

$$S_{11} = S_{22} = \frac{i}{2}(\frac{1}{z} - z)\sin(nkd)$$

S_{11} and S_{21} can be inverted to find n and z in terms of the scattering parameters as follows:

$$\mathrm{n} = \frac{1}{kd}cos^{-1}[\frac{1}{2S_{21}}(1 - S_{11}^2 + S_{21}^2)]$$

$$Z = \sqrt{\frac{(1 + S_{11})^2 - S_{21}^2}{(1 - S_{11})^2 - S_{21}^2}}$$

The Lumerical FDTD software provides a library of ready-to-use S-parameter analysis groups to facilitate the inversion of S-parameters to obtain the equivalent refractive index and equivalent impedance of the meta-structure.
